# [Retracted] Crocin promotes apoptosis of human skin cancer cells by inhibiting the JAK/STAT pathway

**DOI:** 10.3892/etm.2026.13211

**Published:** 2026-06-15

**Authors:** Gongfeng Wang, Baofang Zhang, Yanyan Wang, Shunli Han, Chenghong Wang

Exp Ther Med 16:5079–5084, 2018; DOI: 10.3892/etm.2018.6865

Following the publication of the above paper, it was drawn to the Editor's attention by a concerned reader that certain of the Edu staining assay data in Fig. 1D, the flow cytometric assay data in Fig. 2A, and western blot data in Figs. 3 and 4A were strikingly similar to data that had already been published in other articles in different journals that were written by different authors at different research institutes, one of which has since been retracted.

Upon independently analyzing the data in this paper, the Editorial Office were able to verify the reader’s concerns. Owing to the fact that the contentious data in the above article were found to be strikingly similar to data that had already been published elsewhere in other papers in different journals, the Editor of *Experimental and Therapeutic Medicine* has decided that this paper should be retracted from the Journal. The authors were asked for an explanation to account for these concerns, but the Editorial Office did not receive a reply. The Editor apologizes to the readership for any inconvenience caused.

